# Ureteroscopic management of asymptomatic and symptomatic simple parapelvic renal cysts

**DOI:** 10.1186/s12894-015-0042-5

**Published:** 2015-06-06

**Authors:** XiaWa Mao, Gang Xu, HuiFeng Wu, JiaQuan Xiao

**Affiliations:** Department of Urology, The Second Affiliated Hospital of Zhejiang University School of Medicine, Hangzhou, P.R. China

**Keywords:** Parapelvic renal cyst, Flexible ureterscopy, Holmium laser

## Abstract

**Background:**

To investigate feasibility and safety of treating simple parapelvic renal cysts using flexible ureteroscopy with the Holmium laser.

**Methods:**

Between February 2010 and July 2013, a total of 21 patients, aging from 29 to 71 (49.00 ± 13.23), were diagnosed with parapelvic renal cysts by ultrasonography in combination with contrast-enhanced computer tomography (CT) and intravenous urography (IVU) in the Department of Urology Surgery, People’s Hospital of the Zhejiang province. Fifteen patients were asymptomatic and 6 patients were symptomatic with flank pain. All patients underwent drainage of the cysts using flexible ureteroscopy with Holmium laser. Patients were followed up 1, 3 and 12 months after the operation.

**Results:**

The intervention was successful in 20 patients and failed in 1 patient who, subsequently successfully underwent a laparoscopic cyst removal. There were no intra-operative and post-operative complications reported. The mean operation time was 27 min (range: 15 to 45 min). The mean hospital stay was 2.6 days (range: 1 to 5 days). Twenty patients were followed up until 15 months after surgery. After such ureteroscopic management, there were no renal cysts detected in 7 patients (35 %) and a reduction in size of the renal cysts was found in 13 patients (65 %). Flank pain subsided in all 6 (100 %) previously symptomatic patients.

**Conclusions:**

Flexible ureteroscopy with the Holmium laser may be a feasible and effective treatment option in selected patients with simple parapelvic renal cysts. Further prospective randomized studies that compare the procedure to laparoscopic treatments are needed.

## Background

At least 20 % of adults will have formed simple renal cysts by the age of 40 and up to 33 % will have developed renal cysts at the age of 60 [1]. Most parapelvic renal cysts are found by chance and are generally symptomless but as they are closely associated with the hilar vessels and the collecting system they can produce symptoms of renal obstruction. If symptomatic, the most commonly reported symptoms are lumbar discomfort (followed by urinary tract infection (9.5 %) and hematuria (4.8 %) [2]. Other possible consequences of parapelvic cysts include stone formation, vascular compression, renin-mediated hypertension and spontaneous hemorrhage. A successful diagnosis can be achieved by ultrasonography and contrast-enhanced computer tomography examinations [3].

The management of parapelvic renal cysts has evolved over the past decades from open exploration for marsupilation or nephrectomy to laparoscopic renal-sparing cyst excision, unroofing, decortication and ablation [4, 5]. Percutaneous aspiration and sclerotherapy are contraindicated for parapelvic cysts since potentially serious complications such as extravasation of sclerosing agents from the renal cyst into the retroperitoneum may lead to local inflammation and consequent ureteropelvic junction obstruction, fever, pain and high possibility of cyst recurrence [6, 7]. Successful endoscopic management of renal cysts by antegrade percutaneous nephroscopic ablation and retrograde flexible ureteroscopy have recently been reported [8–10]. The retrograde approach is effective and has a low complication rate. Other benefits include its minimally invasive nature and a short hospital stay after surgery. Basiri et al. reported the successful ureteroscopic treatment of parapelvic renal cysts in 2 cases, while they could not report the long-term follow-up details [9]. In a recent study by Luo et al., treatment of renal parapelvic cysts with a flexible ureteroscope could make 10 of 15 patients cyst free after 6 or 12 months [11]. Despite these promising results, there have only been very few reports describing the management of renal cysts with retrograde flexible ureteroscopy. Furthermore, little information can be found regarding the long-term results of this treatment. The objective of our study was to report the long-term results of management of renal cysts with retrograde flexible ureteroscopy using holmium laser.

## Methods

Between February 2010 and July 2013, 21 patients from the Department of Urology of People's Hospital of Zhejiang Province participated in this investigation. Patients included in this study met the following criteria: presence of a simple renal parapelvic cyst greater than 3 cm in size; no history of ureteral stricture; and presence of a parapelvic cyst wall close to the pelvic wall. Those whose cysts were suspected for malignant in imaging and those who were less than 18 years old or more than 80 years old were excluded from the study. A total of 15 patients were asymptomatic and the cysts were incidental findings during routine medical examination. Six patients were symptomatic with flank pain. Patients were informed in detail about the ureteroscopic operation and possible complications and all patients provided written informed consent before enrollment in the study. Urine analysis, urine culture, serum electrolytes, ultrasonography, intravenous urography (IVU) and contrast-enhanced computed tomography (CT) were performed in all patients prior to the operation. We defined simple parapelvic renal cysts as circular renal cysts, filled with clear fluid and without any connection to the pyelocaliceal system. Ureteral stenting was performed 2 weeks before the operation in each patient to facilitate ureteroscopic access. All data was collected in a prospectively maintained database. Patients were invited for follow-up examinations at 1, 3 and 12 months post-operation. The Ethics Committee of People’s Hospital of Zhejiang Province had agreed to the study.

**Fig. 1:**
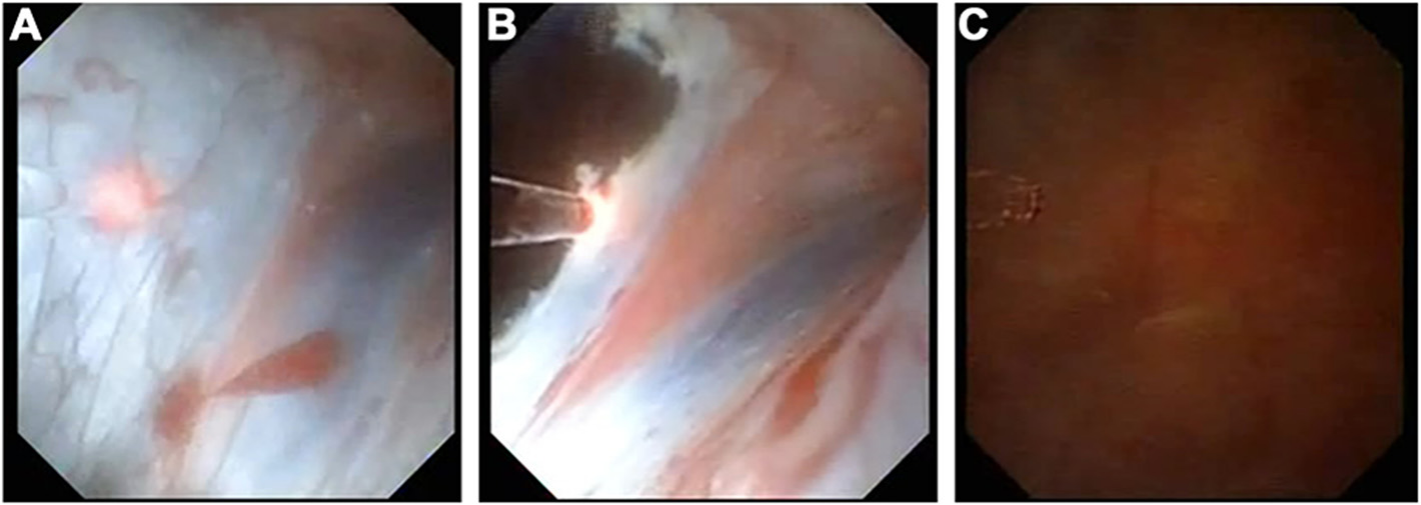
Video screenshots flexible ureteroscopic surgery with Holmium laser for a simple parapelvic renal cysts. **a** A rigid diagnostic ureteroscopy with an 8.5 F instrument is used to dilate and visualize the ureter. **b** A flexible ureteroscope is advanced into the renal pelvis to identify the location of peripelvic cyst. **c** A holmium laser with a 200 um fiber is used to incise and drain the renal cyst wall

The procedure was performed while the patient was under general anesthesia in lithotomy position. All patients were given a prophylactic dose of cefatriaxone (1.0 g) 30 min before the operation. Initially, a rigid diagnostic ureteroscopy with an 8.5 F instrument was performed to dilate and explore the ureter in order to confirm a normal ureter (Fig. 1a). Then, a 12 F ureteral access sheath was introduced over a guidewire. Afterwards, the flexible ureteroscope was advanced into the renal pelvis to identify the location of peripelvic cyst (Fig. 1b). Incision and drainage of the renal cyst wall were performed using a holmium laser with a 200 um fiber (Fig. 1c). If stones were present, lithotripsy was performed during the same session. A window in the cyst as large as possible was opened with a holmium laser (Lumenis, VersaPulse®, PowerSuite™, 60 W, two wavelength for adequate drainage. The laser setting were 0.8 J and a frequency of 25 Hz, a combination which is less likely to injure adjacent tissue we find more accurate. Finally, a 6double J stent was placed in the ureter with upper end inside the cyst… The stent was removed one month after the operation.

Patients’ demographic characteristics,, the operative time, surgical time and length of hospital stay were recorded. The cyst size was measured before the operation, and during the follow-up period in an outpatient clinic at 1, 3 and 12 months after the surgery. The follow-up examination included a CT scan and ultrasound. Lack of detection of the cyst or a decrease in size to less than 50 % of the original mass was considered evidence of a successful operation [12].

## Results

Patients' basic characteristics are detailed in Table 1. A total of 21 patients underwent flexible ureteroscopic drainage of simple renal parapelvic cysts using a holmium laser. The patient population included 12 males and 9 females and the mean age was 49 years (range from 29 to 71 years). Additional patient details are presented in Table 1. The mean cyst size was 4.5 cm (range from 3 to 7 cm) and stones were found inside the renal cysts (mean size 0.8 cm) of 3 patients.

**Table 1:** Patients’ characteristics

Patient number	Gender	Age	Preoperative cyst size (cm)	Surgical time (min)	Hospital stay (day)	Cyst size at 1 month after the surgery (cm)	Cyst size at 3 months after the surgery (cm)	Cyst size at 1 year after the surgery (cm)
1	Male	35	4	24	2	1	0.5	0.5
2	Male	41	5	30	2	2	2	1
3	Male	29	4	33	1	2	1	1
4	Male	59	6	15	3	3	2	1
5	Male	68	4	30	4	2	2	2
6	Male	26	5	18	2	2	2	2
7	Male	43	4.5	22	2	0	0	0
8	Male	47	4	22	4	0	0	0
9	Male	29	3	25	2	0	0	0
10	Male	70	4	45	5	2	2	1
11	Male	38	3	24	1	1	1	1
12	Male	54	5	23	4	2	2	0
13	Female	62	3	20	2	0	0	0
14	Female	71	4	37	2	0	0	0
15	Female	33	4	29	3	1	1	0
16	Female	41	4	27	4	0	0	0
17	Female	45	6	35	2	3	2	0
18	Female	56	7	30	2	2	2	0
19	Female	60	3	34	3	0	0	0
20	Female	50	7	17	2	3	2	0
21	Female (switch to laparoscopic surgery)	72	5	59	3	0		

The ureteroscopic procedure was successful in 20 patients with no major complications and no trauma to adjacent organs and great vessels; no pneumothorax or hemothorax were detected. The mean operation time was 27 min (range: 15-45 min) and the mean hospital stay was 2.6 days (range: 1-5 days). One operation failed as the renal cyst wall could not be found endoscopically. The procedure was converted to laparoscopy which made renal cyst unroofing possible.

Postoperativ fever was noted in one female patient 2 h after the operation (38.5 °C temperature, with a low blood pressure 85/65 mmHg). The patient’s parapelvic renal cyst contained tens of small stones with sizes ranging from 2 to 4 mm and the pre-operative urine culture had demonstrated the presence of Escherichia coli. After antibiotic treatment, the urosepsis settled in 3 days.

**Fig. 2:**
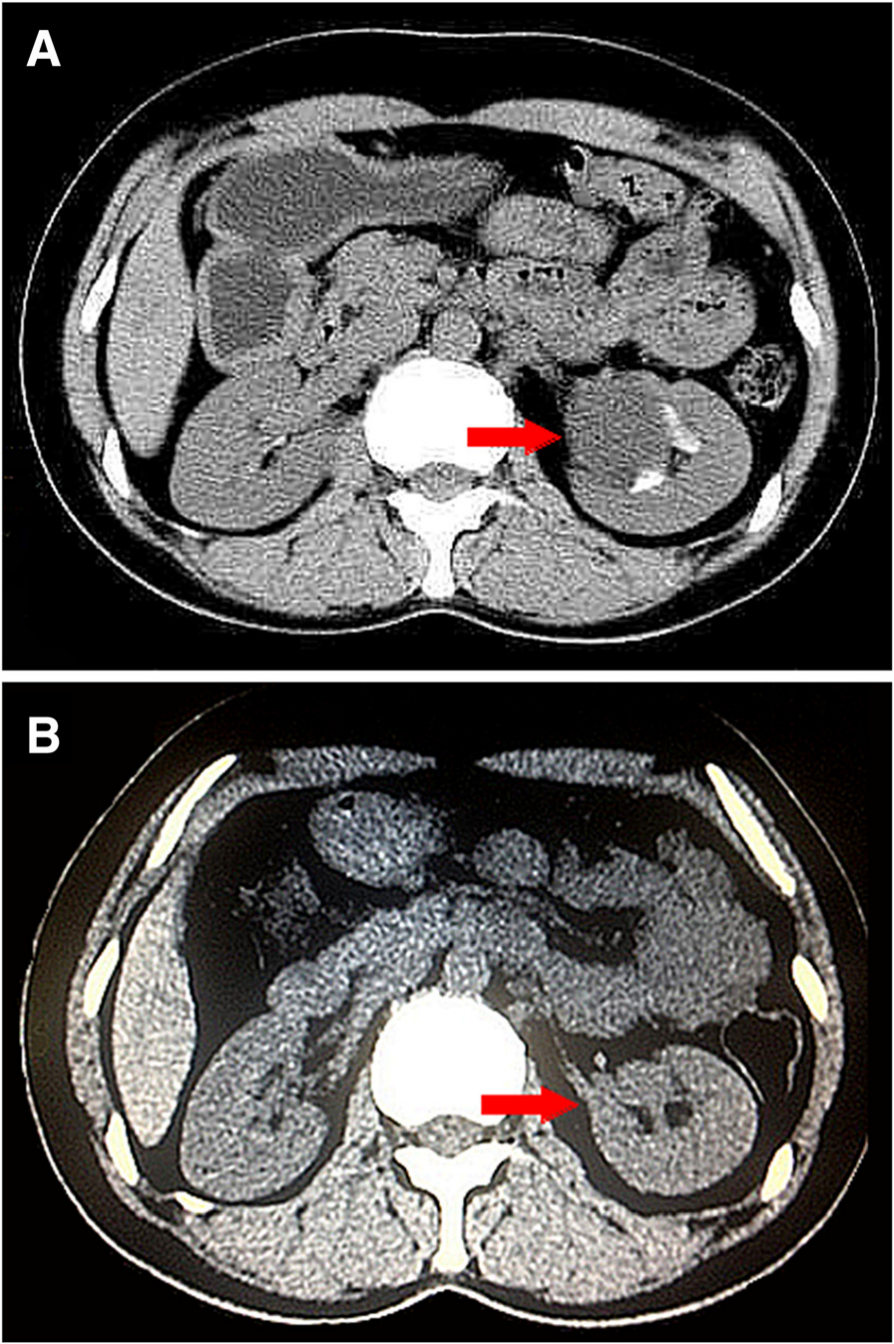
The CT scan showed the parapelvic renal cyst before (Fig. 1**a**) and 1 month after the operation (Fig. 1**b**) in a patient (male, 56 years old). The location of the cystis marked by arrows

Patients underwent follow-up examinations at 1, 3 and 12 months post operation. One month after the operation, there was no evidence of renal cysts in 7 (35 %) patients and 13 (65 %) patients experienced a mean reduction in cyst size of 49.2 ± 12.6 mm to 20.0 ± 7.1 mm. The mean size of the renal cysts of the 13 patients further decreased to 16.5 ± 5.5 mm 3 months after the operation. The renal cysts of 5 patients were no longer detected 12 months after the operation, indicating no detection in 12 patients in total; and the mean size of the renal cysts in the remaining 8 patients further decreased to 11.9 ± 5.3 mm 12 months after the operation. Patients’ outcomes are detailed in Table 1. Complete resolution of flank pain occurred in all 6 (100 %) previously symptomatic patients. A typical CT scan of a non detectable renal cyst 1 month after the operation (Fig. 2).

## Discussion

The terms parapelvic and peripelvic cysts generally describe cysts around the renal pelvis or renal sinus [13]. They do not communicate with the collecting system and are believed to be lymphatic in origin secondary to obstruction. Generally, most of renal cysts are symptomless, but they can occasionally cause lumbar discomfort, haematuria, hypertension and hydronephrosis. On IVU, parapelvic renal cysts can show stretching and compression of calyces, similar to the appearance of renal sinus lipomatosis which involves proliferation of sinus fat leading to a mass effect on the intrarenal collecting system [14]. CT scan does not show enhancement following the administration of intravenous contrast dye and there is usually no hydroureter. On ultrasounds those renal cysts are generally centrally-placed and may be mistaken for hydronephrosis. Therefore, imaging with contrast dye in form of an IVU or a CT urogram should be performed for differential diagnosis.

Until now, various treatments of simple renal cyst have been proposed with varying outcomes, including sclerotherapy, laparoscopic unroofing and percutaneous ablation [8, 15, 16]. Compared with the treatment of simple renal cyst, the treatment of parapelvic renal cysts can be more difficult because of the location near the renal hilum and renal vessels. Sclerotherapy is considered to be contraindicated in the treatment of parapelvic renal cyst because it can provoke perirenal inflammation and subsequently, ureteropelvic junction obstruction. Laparoscopic unroofing requires advanced surgical skills, is considerable invasive and unavoidably leads to comparatively more blood loss. However, it is a common treatment for cysts which are not parapelvic or those who are not suitable for ureteroscopy for example because of ureteral stenosis. Percutaneous ablation has results similar to laparoscopic treatment and has better results than aspiration with or without using sclerosing agents. This from of treatmen however, is invasive and requires the help of radiologist in the operating room.

Liaconis and Basiri first reported ureteroscopic treatment of parapelvic renal cyst with flexible and semirigid ureteroscopy [9, 10]. Both were preliminary studies with only one or two patients and a short follow-up period (3 and 6 months). Luo et al. reported disappearance of parapelvic renal cyst in 10 of 15 patients at 6 or 12 months after flexible ureterescopy [11]. To our knowledge, the current study evaluating the efficacy of ureteroscopic treatment of parapelvic renal cyst included the largest number of patients with a relative long follow-up. Compared with other methods, management of parapelvic renal cysts by flexible ureteroscopic with a holmium laser is characterized by its minimally invasive nature and a low complication rate. A major disadvantage of the treatment is that it cannot provide a pathological specimen. This suggests that a laparoscopic operation should be considered in patients with complicated parapelvic renal cysts that may be malignant in order to achieve an accurate pathological diagnosis.

The key point of this operation is to identify the renal cyst wall in order not to injure the renal parenchyma or renal vessels. The typical renal cyst looks transparent and is black and blue in some areas injecting methylene blue into the cyst can help the surgeon to identify the cyst wall more accurately. We encountered a low complication rate. In our study urosepsis was found in one patient with a parapelvic cyst containing stones. This finding implies that special attention must be paid to such patients. In addition, it should be noted that one operation failed in our study due to the difficulty in identifying the wall of the renal cyst. In the study by Luo et al., the mean total operation time, and mean duration of hospitalization were 31 ± 8 min and 3 days, respectively [11]. In the present study iopertive time was 27 min (range: 15 to 45 min) and hospital stay was2.6 days (range: 1 to 5 days), respectively.

Apart form a short operative time and a short hospital stary, retrograde intrarenal surgery may have further advantages compared with laparoscopic unrrofing: A possible serious complication of laparoscopy is formation of a urinary fistula due to inability to differentiate a parapelvic renal cyst and the renal collecting system. Moreover, laparoscopy has shown to have a greater significant estimated blood loss, increased operative time, and increased length of hospitalizationas opposed to the truly mini-invasive retrograde intrarenal surgery. Limitations of our study were that we had no comparison group and that a longer follow-up would be beneficial.

## Conclusion

Flexible ureteroscopic treatment of parapelvic renal cyst is a feasible and effective treatment for parapelvic renal cysts. Compared to previous similar studies, this report represents the largest number of patients with the longest follow up period. A large prospective randomized study comparing this procedure to laparoscopic operation is warranted to confirm these results.

## Abbreviations

CT: Computed tomography
IVU: Intravenous urography
